# A survey of Canadian medical physicists: software quality assurance of in‐house software

**DOI:** 10.1120/jacmp.v16i1.5115

**Published:** 2015-01-05

**Authors:** Greg J. Salomons, Diane Kelly

**Affiliations:** ^1^ Department of Physics Queen's University Kingston ON; ^2^ Department of Medical Physics Cancer Center of Southeastern Ontario Kingston ON; ^3^ Department of Mathematics and Computer Science Royal Military College of Canada Kingston ON Canada

**Keywords:** radiation therapy, guidelines, software, quality assurance, testing

## Abstract

This paper reports on a survey of medical physicists who write and use in‐house written software as part of their professional work. The goal of the survey was to assess the extent of in‐house software usage and the desire or need for related software quality guidelines. The survey contained eight multiple‐choice questions, a ranking question, and seven free text questions. The survey was sent to medical physicists associated with cancer centers across Canada. The respondents to the survey expressed interest in having guidelines to help them in their software‐related work, but also demonstrated extensive skills in the area of testing, safety, and communication. These existing skills form a basis for medical physicists to establish a set of software quality guidelines.

PACS number: 87.55.Qr

## I. INTRODUCTION

The field of radiation therapy has been rapidly evolving since its inception, and the medical physicist's role has been both to drive that evolution and to ensure that it is done safely.^(1)^ Commercial software, by its nature, lags behind needs of practice since it takes time to respond to the demands of the customers. In addition, since methods and procedures can vary substantially amongst clinics, commercial software developers face a huge challenge in producing software that will fit the requirements for all clinics. Often commercial developers choose to write software solutions that they believe to be most efficient or the most common and ignore all other scenarios deemed less profitable. As a result, medical physicists have sometimes found it necessary to write their own (in‐house) software to deal with new technology or scenarios that commercial companies do not feel are profitable to address.^2^, ^3^

The physicist who writes software must also ensure that the software is safe and used in a safe manner.^(4)^ As a result, some have questioned the legitimacy of in‐house software, arguing that any software used clinically must be approved by a regulatory body before it is appropriate to use clinically. However, in both Canada and the USA, the regulation of medical devices by Health Canada or the FDA applies only to commercial products.^5^, ^6^ Some have also pointed to ISO 13485 as a source of guidance for software quality, but again this standard is directed at the manufacturing industry and is ineffective at providing guidance on safe software. For example, ISO 13485 requires mandatory purchase control and environmental control, but design activities are considered optional.

While it is clearly within the purview of the medical physicist to develop spreadsheets and more complex software for clinical use, the AAPM does not have any task groups addressing software development. It does have some task groups that address the functionality of specific types of software. For example, Task Group 201 is charged with recommending “Quality Assurance of External Beam Treatment Data Transfer” and Task Group 53 was changed with Quality Assurance of Treatment Planning Systems. None of the relevant international bodies, such as the ICRP, address the issue of in‐house development. Since there are no established guidelines for software development and software quality assurance (SQA) in the medical physics discipline, it has been up to individual physicists and physics departments to determine how this is done.

The authors of this paper sent out a survey to cancer centers across Canada to assess the extent of in‐house written software and the desire and/or need for software quality guidelines. For the purposes of this survey, we defined “in‐house” software as noncommercial software, expressed as scripts, spreadsheets, or in programming languages, written by medical physicists or other technical staff, for use at the clinic where it was written or for limited free distribution.

The medical physicists who responded to this survey described a wide variety of software practices that are focused on assuring their software is trustworthy and used safely. These local practices have been developed specifically for the medical physics safety context. These practices are already in use and, if compiled, have the potential to provide the groundwork for establishing wider guidelines for the creation and use of in‐house medical physics software. The survey gathered both respondents’ perceived concerns and respondents’ detailed descriptions of current software quality practices. This provides a picture of perceived weaknesses and actual strengths. The strengths are useful lists of existing practices suitable for generating software quality guidelines. Identified weaknesses can eventually be addressed by looking at practices from other suitable scientific domains.

This is the focus of this paper: we discuss the results of the survey in terms of software practices within and without the medical physics community, and suggest steps in disseminating established local practices to a wider audience.

## II. MATERIALS AND METHODS

### A. The survey

In the fall of 2012, the authors of this paper distributed a survey and accompanying email to people primarily at cancer centers across Canada. The purpose of the survey was explained in the email as “intended to explore the practices and policies employed by medical physicists in the development and use of in‐house software for clinical purposes”. The point of the survey was to explore the question of whether safety‐oriented software practices existed in the community. If these practices exist, then they provide a basis for the community to educate itself. One of the ways of educating itself is sharing the existing safety practices by compiling SQA Guidelines based on those existing, realistic practices.

The survey consisted of three parts. Part 1 contained eight multiple‐choice questions with text space available to expand answers. These questions covered the utilization, maintenance, and documentation of in‐house software in the respondent's local clinic. The question in Part 2 asked the respondents to rank the need for policies or guidelines related to a list of suggested SQA topics. Part 3 of the survey consisted of seven questions allowing free text comments on testing and QA practices being applied to new and modified software. Part of the survey lent itself to statistical analysis. The free text comments allowed a qualitative analysis of the breadth of software practices already in existence in the medical physics community.

The survey was carried out under the auspices of the Research Ethics Board (REB) of the Royal Military College of Canada. Respondents were told that their participation was voluntary and that confidentiality would be maintained.

The invitation to participate in the survey was sent out by email to 397 people in October 2012 and to 22 additional people two weeks later. The invitations primarily targeted medical physicists working at cancer clinics within Canada; however, 11 invitations were sent to medical physicists working in Canada but outside the cancer clinic environment. A further 11 invitations were sent to medical physicists working outside of Canada who had ties to the Canadian medical physics community. The closing date for the survey was November 2012.

In Canada, health care is regulated by each province, with the number and size of the cancer clinics in each province depending on the population density and the model of care delivery employed in that province. Often, but not always, the cancer center is affiliated with a hospital that has an active medical school. A recent survey indicated that a typical cancer clinic has five to ten physicists, with a case load of 260 new patients per physicist per year.^(7)^

In total there were 48 respondents to the survey, two of which did not complete the multiple choice sections and were eliminated from the analysis. The remaining surveys correspond to an 11% response rate. Of the 46 completed responses, 44 were from across Canada and one each from Australia and the United States. There were 24 responses from Ontario (from 8 out of 16 cancer centers), 6 from Manitoba, 4 each from Alberta and Quebec, 2 each from Nova Scotia and British Columbia, and 1 each from New Brunswick and Saskatchewan. No responses were received from either PEI or Newfoundland. The large number of medical physicists in Ontario and Quebec constituted 65% of the total responses. The responses provided sufficient sampling to paint a broad picture of the software practices present in the Canadian medical physics community.

To analyze the eight questions of Part 1, responses were grouped by site using IP address. This was possible since the surveys were completed on computers located behind hospital firewalls so that IP addresses were unique to each site and not to individuals. The grouped responses were averaged for each question and a single averaged response was assigned for each site so that sites with many respondents would not bias the results.

Each question had “other” as a possible response, with room for free text. It appears that some respondents used “other” when they were unclear about which choice to select. Where appropriate, during analysis, “other” was reassigned to a different selection based on the descriptions included in the free text comments.

## III. RESULTS

### A. Part 1: Current practice

Table 1 gives a statistical summary of the responses to the questions in Part 1.

According to the survey, assuring the quality of radiation treatment is the most prevalent use for in‐house software, with more than 70% of the sites reporting that they use in‐house software for some type of radiation quality assurance purposes. Sixty‐five percent (65%) reported using their own software to track or record processes. Thirty‐five percent (35%) reported using in‐house software as part of patient specific QA, and less than 20% of respondents reporting that they use in‐house software for treatment planning or treatment delivery.

The respondents reported using a wide variety of in‐house software types, with complexities ranging from simple spreadsheets to large complex code. The comments accompanying this question indicate that the programming languages used are varied in nature, ranging from Visual Basic scripts inside office documents through high‐level languages (such as MATLAB and C++) and low‐level assembly languages.

The responses indicate that in‐house software is never used by the author alone, and in only 28% of the responses, in‐house software use is limited to the physicists. Fifty‐five percent (55%) of the responding sites reported in‐house software use that extends beyond the physics department, and 37% reported multiple departments using in‐house software. In designing the survey, it was assumed the author of the software would use his/her own software and share that software with others. However, comments accompanying the responses indicated that, in a few cases, software is written for other users and not used by the author at all.

**Table 1:** The percentage of responses for each question in Part 1 of the survey. Percentages represent proportion of responses by site rather than by individual. Unless otherwise indicated, questions asked respondents to select all choices that applied to them

	*Response*	*Percentage*
1. What clinical tasks is the in‐house software at your center used for?	Record Keeping	71%
	Dose Calculation QA	78%
	Machine QA	82%
	IMRT QA	33%
	Dose Planning or Delivery	18%
	Other	0%
2. What types of in‐house software is being used at your center?	More complex code	84%
	Short scripts	71%
	Spreadsheets	93%
	none	0%
	Other, please specify…	0%
3. Who uses in‐house software at your center? (select one)	Multiple departments	40%
	Dosimetrists	20%
	Physics department	20%
	Physicists only	16%
	Author only	0%
	Other	4%
4. What steps are taken to prevent inadvertent modification of the software or spreadsheets?	Protected cells in spreadsheets	83%
	Read only access	57%
	User Privileges	55%
	Compiled Software	52%
	Other	7%
5. What methods are used to archive old versions of the software or spreadsheet?	Copies of older versions	93%
	Version Control Software	33%
	Test data and results are kept	30%
	Permanent off‐line storage	7%
	Other	5%
	None	2%
6. What training is generally given to staff before they begin to use your in‐house software?	Peer Instruction	79%
	Written documentation	44%
	Formal training	40%
	Self Directed	33%
	Mix of methods	21%
7. What documentation is generally made available for in‐house software or spreadsheets?	Comments in code	52%
	Little if any	26%
	A user's manual	26%
	Other	19%
	A “Readme” file	17%
8. How formal are your procedures for the use and creation of in‐house software? (select one)	Up to Author	35%
	A General Understanding	40%
	Written Guidelines	7%
	Strict Policy	2%
	Depends on Users	16%

The results to the fourth question indicate that most centers use at least one method to prevent accidental modification of software or data. In addition to the options given in the survey question, respondents also listed checksum tests, password protection, and controlled access. Three respondents reported that there is a general understanding that only the author may modify software and data.

According to Question 5, almost all clinics keep older versions of software, though in the comments some indicated that they only keep the previous version and not all versions.

Only one respondent stated that they keep no records of previous versions. One‐third of the respondents indicated that they use a version control system. In addition, a number of people indicated that they are considering or interested in software to aid in version control, but have not yet implemented it. Only 30% of the respondents reported keeping old test data and results.

Question 6 asked, “What training is generally given to staff before they begin to use in‐house software?” The most common method is one‐on‐one training, or mentoring. Some respondents indicated that the type of training given depends on who are the users of that particular software. Others indicated that training is a combination of written material and peer instruction. One‐third of the respondents indicated that software training of users is primarily self‐directed. The comments indicated that training on the use of the software is mostly linked to training for the specific task for which the software is designed.

Question 7 was about the types of documentation available. Three‐quarters of the respondents specified at least one type of document. Code comments were listed by more than half of the respondents. Slightly less than half included user manuals and “readme” files. Other types of documentation included peer reviewed publications, policies and procedures, and wiki documentation.

Question 8 asked about the formality of procedures for use and creation of in‐house software. Less than 20% of the sites reported any formal policy or written guidelines, and more than 30% indicated that software quality is the responsibility of the author of the software. However, nearly 70% of the respondents indicated that they have at least an informal, if not formal, “general understanding within the department about how things should be done”.

### B. Part 2: Ranking software topics for QA concerns

This question on the survey asked the respondents to rank in order from most important (1) to least important (9) a list of activities that could (or should) be addressed by software guidelines.

**Figure 1:**
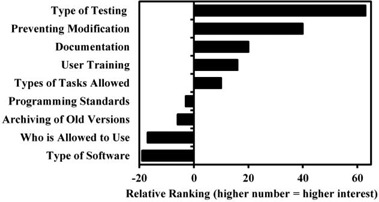
Relative interest in guideline.

The responses to this question were not grouped by site, as was done for questions in Part 1, since this question was reporting individual respondent's opinions rather than conditions in the clinic. There were 42 responses to this question. Of these, 12 responses did not clearly differentiate a ranking for at least 50% of the activities listed; for example, one response ranked everything as 4 or 5. These 12 responses were removed from the counts. From the 30 remaining responses, we have the results, as shown in Fig. 1.

The greatest concern shown by respondents is testing in‐house software prior to clinical use. Of the next greatest concern is preventing inadvertent modification of the software. Third and fourth are related: appropriate documentation and user training. Of least concern is mandating who can use the software and what types of software are allowed to be used.

### C. Part 3: QA activities in more detail

Seven free‐text questions asked about activities used to check software before being put into clinical use. Answers generally were detailed and provide a rich picture of the activities across the cancer clinics. Below each question, we provide a summary of responses.

1. Who usually finds errors or defects with the software? How are they reported? How are they dealt with?
  Most responses included the end‐user as a person who finds defects in the software. Reporting of defects is most often directly back to the software author. The author of the software is also listed as a key detector of defects, as well as several different participants in the development process, such as first test users, those involved in the implementation phase, the physicist examining results from the software, those doing beta testing before clinical use, independent testers, physicists typically testing the program offline before use, physicists as part of rigorous testing prior to clinical release, and a test group. Mostly, the software author makes the changes as necessary. In some cases, the software will be pulled from service until the changes are done. In other cases, users will be notified of the problems. In a few cases, fixes are batched, depending on their severity and a new version of the software is released. In rare cases, fixes are discussed by a committee.
2. If your center receives source code for a piece of software, what activities do you carry out before putting the software into clinical use?
  All those who received source code from others talked about testing code to increase confidence in it. The testing approaches described were varied and thoughtful, with most respondents describing more than one testing approach. The testing approaches fall roughly into four categories: validate the software against known solutions, whether the solutions are software generated or calculated by hand; set up specific scenarios such as clinically relevant situations, edge cases, or incorrect data and observe the behavior of the software executing those scenarios; use the software “for reference only” in clinical situations with real users and monitor its behavior; verify that the software code conforms to equations and coefficients by reading the code.
3. If you write a piece of software intended for clinical use, what activities related to the development and implementation of that software do you feel are the most important to ensure the answers from the software are correct?
  A variety of activities to check software were listed, covering the range of software development phases. Early phases of software development include carefully thought‐out software designs and determining details of software requirements and the problem domain. A number of responses mentioned developing a test plan in advance and testing early in the development of the software. Only one comment mentioned ISO certification: “commercial grade quality system for unit testing, validation, and verification”. One other respondent mentioned QA audits. Several responses mentioned having someone else involved (e.g., at least one other physicist, the users, beta testers, third‐party checkers, trial periods running in parallel, and independent testers).
  One person noted that: “If you don't fully comprehend the algorithm and its limitations, you won't be able to meaningfully interpret the data”. In addition, one respondent noted that the test results must be investigated until they are understood, and another mentioned observing end‐user's performance to look for program weaknesses, ease of use, etc.
4. If you are working with an existing piece of in‐house software, what sources of information best help you understand the details of how the software works?
  Two sources of information that were mentioned most (43% of respondents) were: (i) talking to knowledgeable people such as the author of the software and experienced users (most of the time, it was the software author who was mentioned); (ii) reading documentation, especially user manuals in various guises, but also programmers’ notes and manuals, commissioning reports, and upgrade notes. Next most frequently mentioned (24%) was reading source code and embedded comments. Department protocol was mentioned by 9% of the respondents as a source of information, followed by textbooks (7%), and online user group (4%).
5. If you are creating or modifying a piece of in‐house software, what do you do to ensure someone else or you can understand the details of how the software works several months or several years later?
  The largest proportion of respondents (42%) stated they wrote in‐line comments in the code to assure future understanding. Next most frequently (22%) described by the respondents was a “how to use” document, whether it is a user manual, read‐me file, or instructions in a graphical user interface. Articles on the theory, including peer‐reviewed papers, were mentioned by 14% of the respondents. Also mentioned were evaluation reports, which include assessments for precision, user's feedback on use, and “independent” reports; documentation of the internals of the program, beyond in‐line comments — for example, descriptions of data structures, limitations of the software, and reasons for implementation and theory choices. Revision histories and bug reports, describing sample input/output and updating department policies were mentioned by less than 10% of the respondents.
6. Are there software rules in place at your center regarding the creation and modification of in‐house software? If so, would you be willing to provide us with a copy of these policies or guidelines?
  The lack of formally written rules or policies resulted in no offers of copies of such. However, comments reflected (as did previous questions) the existence of informal procedures that included checking with others, committee reviews, limited distribution of software, and central management of changes.
7. Please feel free to add further comments regarding useful software practices or the value of standardized guidelines.
  The responses to Question 7 reflected responses to previous questions. Particularly, there were comments to the effect that general guidelines would be welcomed, that “the questionnaire was useful in stimulating thought and discussion”, that guidelines have to recognize the variability in the medical physics environments, and the imposition of guidelines have to recognize the limited human and financial resources available.

## IV. DISCUSSION

### A. Context

The first three questions in Part 1 provide a picture of the prevalence and usage of in‐house software. The context in which software is used has significant implications for the steps required to ensure its safety. This context includes what the software is used for, who is using it, and the nature of the code or software platform.

The vast majority of the software is used for medical QA purposes. Such software has an inherent safety factor built into its use in that errors in the software or in its application will not directly cause harm to a patient. There are two primary quality concerns for software used in medical QA applications. The first is a false negative, where the software fails to identify a fault in the system it is intended to be testing. The second concern is false positives, where the software incorrectly indicates a fault in the system it is testing. An occasional false positive is usually only a matter of inconvenience, since alternative methods of testing the system usually exist. However, a large number of false positives can have the effect of negating the value of the medical QA procedure since human nature will inevitably cause the fault warnings to be ignored. As a result, evaluation of the appropriateness of the testing algorithm is an important component of software safety.

The developer/user profile of the software covers a variety of scenarios, from code developers sharing software with other medical physicists, sharing with other departments and sometimes multiple departments, and writing software specifically for others without the developer being a user. The survey was written assuming single person authorship of individual code and the comments received supported this assumption. There were no cases where the code developer was the only user of the software. Groups of people, rather than single individuals, are involved with each piece of software. Responses in later parts of the survey indicate extensive interactions within these groups, with peer‐to‐peer training being the most common method of communicating information about a piece of software.

Software types were quite varied, with almost everyone using spreadsheets. Comments in the survey and reports from other industries indicate that some people do not recognize spreadsheets as a form of programming. Experience, both in the medical physics community and in other industries, indicate that software safety principles apply to spreadsheets just as much as to any other type of programming.^8^, ^9^ General guidelines already exist for writing “good” spreadsheets, and the medical physics community can borrow extensively from this to generate appropriate spreadsheet guidelines.

The definition of “short script” versus “more complex code” was left up to the respondent. In this way the responses were linked to the programming experience of the respondent, allowing them to rank the code they are involved with according to their idea of what makes it complex. More than 80% reported using what they considered “more complex code”. In addition, a large variety of software languages were reported being used.

### B. Software integrity

The fourth and fifth questions in the survey explored activities done to ensure the integrity of the software over time (i.e., that the software and corresponding data have not been accidentally modified and that it is possible to compare the present state of the software and data with older versions). The results indicate that, overall, the medical physics community is aware of this issue and has taken steps to deal with it.

Almost all respondents reported keeping older versions of software, but only 30% reported keeping old test data and results. This could be related to the purpose of the software in question. For example, software used for recording and trending medical QA data would not likely benefit from the preservation of a test dataset. However, for software that performs calculations, it may be valuable to preserve some test data as part of ensuring its continuing integrity.

Version control software was reported by 30% of the respondents. The respondents also commented that some had a desire to use version control software and are looking for guidance on this. The value of a version control system is likely related to the complexity and type of software being developed, along with the frequency with which modifications are likely to be made.

The responses to these questions showed there is experience in protection and archival activities. However, some centers are operating on a trust‐based system. The experience within the community can be exploited to determine suitable activities for other medical physics centers.

### C. Training & documentation

The sixth and seventh multiple‐choice questions addressed one aspect of communication— what is being done to ensure that users are familiar with the software. The most common method of training is one‐on‐one training, or mentoring. Less than half of the respondents reported using written documentation as part of the training. Since most of the software is being used for medical QA purposes, it is possible that software documentation is being included as part of documentation for the medical QA procedure and was not recognized as software documentation by the respondents. The lack of documentation may also be related to the preferred training approach. One‐on‐one peer or one‐on‐one mentoring is a common training method for complex scientific domains.^(10)^ It may also be that software developers have found that formal documentation has not provided significant added value for the amount of time and effort required to generate and maintain it. Lethbridge et al.^(11)^ found that, “More than one‐half of the respondents [to their survey] found the available software documentation effective when learning a new software system, testing a system, or working with a new system. Fifty percent found it effective when other developers are unavailable or when looking for big‐picture information. Only approximately one‐third of the respondents found the documentation effective for maintaining a system, answering questions about the system, looking for in‐depth information, or working with an established system.” The researchers further cautioned that their research “suggests that documentation use might be even less than reported.” Useful documentation is highly dependent on the situation. Having a community to turn to and knowing where to look for information seem to be the most effective approaches.

### D. Software policy

While very few centers reported having formal policies for software development, most indicated that there is a “general understanding” of how things should be done. This “general understanding” can be recorded and serve as a start to setting up software quality guidelines for each software center. This can also serve as a start for discussions across centers. Part 3 of our survey, in effect, is an indicator of this “general understanding”.

### E. Guidelines

Published guidelines are of little use if the community sees no value in them or does not trust them. The second part of the survey addresses the community's desire for software guidelines. The two issues of least concern are likely very specific to particular clinics. Of wide concern is effective testing. Even though the respondents indicated they most wanted guidelines on testing, their responses in the third section of the survey showed they already pay significant attention to testing. They describe testing methods that are varied and effective. The concern with testing is indicative of the risk‐averse environment of the medical physicists.

Interestingly, programming standards were not ranked highly. This is one area that other scientific domains claim to be extremely important.^(10)^ Advice would be for the medical physicists to include programming standards in any software guidelines, for the sake of ongoing communication and long‐term maintainability. Because of the typical long lifetimes of scientific software,^(12)^ long‐term maintainability is an important software quality for scientists. A dialogue about programming standards with software specialists from other safety‐related scientific and engineering industries may prove valuable.

### F. Testing practices

The respondents to the survey ranked testing of greatest concern for software guidelines. Testing is seen by the software community as a key activity in any software development.^13^, ^14^ However, software practitioners state, “One key to improving the state of software testing is sharing ideas through case studies and lessons learned.”^(14)^ The sharing needs to be focused however, with the recognition that “Testing … can benefit from restricting the scope [of a particular testing method] to the needs of a specific domain.”^(13)^ In other words, software developers in a particular field who have testing methods that work well for their software, have the means of helping others in the same field by sharing their experiences.

The survey showed that there is a wide variety of experience with testing in the medical physics community. Table 2 is a summary of the responses to Questions 2 and 3 in Part 3 of the survey. The responses are organized into six themes apparent from the responses. Individual responses often spanned multiple themes and were assigned to each theme to which they applied. The responses in each theme were then summarized into a number of related elements.

The contents of Table 2 illustrate the breadth of experience held by the medical physics community as a whole. This experience puts the medical physicists in an ideal position to judge and improve their own testing approaches. Bertolino^(13)^ reports, “A more recent review of testing technique experiments [in the software engineering community] sadly concluded that over half of the existing (testing technique) knowledge is based on impressions and perceptions and, therefore, devoid of any formal foundation.” In other words, the medical physics community with its extensive experience in testing its own software has developed a decided advantage in this area.

**Table 2:** Summary of responses regarding testing and documentation from Questions 2 and 3 of Part 3 in the survey, grouped into six different themes, extracted from survey

*Test at different stages*
Start with basic scenarios then more advanced scenarios
Test software at various intermediate stages, not just final result
Have actual users test software
Use the software in the clinic for reference only by a small group of people and monitor its behavior
Evaluate software for ease of use
Follow release of software by a period of frequent QA
*Verify the software against theory*
Evaluate algorithms used to understand the limit of accuracy and any situations where it diverges from reality
Have second physicist read the code and check that equations and coefficients are correct
Determine if differences between software output and other solutions are due to modeling and assumptions or due to bugs in the software
Use the source code as a model to develop a new version of the model and use it to compare
*Use multiple sources of known solutions*
Hand calculations
Clinical measurements
Historical records and data
Previous versions of the same software
Other similar software
*Test multiple scenarios*
Clinically relevant
Edge cases (at limits of clinically relevant parameters)
Unusual situations
Incorrect data
Data out of sequence
*Test strategically*
Design tests to answer a specific question
Consider process and workflow in test design
Identify situations where failure will cause the most harm
*Documentation is important*
Organize the code for easy reading and debugging
Document expected input and output for algorithms and data required to generate the output
Write the user manual as part of the testing
Document, test results
Make sure user knows how to use the software properly
Have procedures for bug reporting, and for software upgrade following bug repair

## V. CONCLUSIONS

The goal of this survey was to examine the need and desire for software quality assurance guidelines within the medical physics community in Canada, specifically for in‐house developed software.

From the survey, in‐house developed software seems to be widespread. However, the types, uses, and characteristics of this software vary considerably, making universal guidelines for the entire community difficult to design. None of the survey respondents said “no guidelines”, but several respondents cautioned there is a need for “recognition that different levels of validation are required for different stratifications of usage and impact”.

If guidelines were to be instituted, results from this survey provide practical suggestions for the way forward.

From the survey, for the implementation and use of in‐house software, nearly 70% of the centers have at least a common understanding within their department on how to develop and use software. Several respondents demonstrated a risk‐averse philosophy in dealing with their software, describing “independent, redundant, and parallel generation of results to reduce risk to the patient, independent of software testing and version control”. This holistic system view is typical of software developed in other scientific disciplines that are risk‐averse.

Universal guidelines based on those promulgated by the software engineering community are process‐based. In other words, the assumption is that following the process will result in a quality product, much like a building a car on an assembly line. Kitchenham and Pfleeger^(15)^ warned that process‐based software quality standards tend to entrench mediocre products, and “this process focus can lead to quality assessment that is virtually independent of the product itself.” Process‐based guidelines do not take the holistic view that is typical of scientific software development. Post and Kendall^(16)^ advise, based on decades of experience with multiphysics software systems, that a set of locally established and proven practices are far preferable to following a fixed process. Vessey and Glass^(17)^ criticize the “one‐size‐fits‐all” approaches commonly used in software engineering, and explain that “strong” solutions depend on developing approaches specific to the given environment.

One respondent to the survey commented that “we need to steer a fine line … making rules so onerous that it becomes impossible to write any [software] and recognizing our own responsibility as physicists when we use such software … we should be using such tools as an aid, not as a substitute for our own judgment”. This ties into the fact that the user of this software is a key component of the system, not part of an assembly line. The process‐based approach to software development essentially removes the skilled human, the scientist, from the picture. In the development of scientific software, the scientist is an important part of the picture, and the survey respondents acknowledged their role. By focusing on practices rather than process, quality software development becomes the responsibility of the scientist rather than the responsibility of “the process”. The respondents to the survey described having holistic views, sharing experiences, and engaging in teamwork. All this underlies success in choosing appropriate practices for software development and maintenance, no matter the type of software.

Apparent from the survey are the differences in the medical physics departments across Canada. This suggests that guidelines cannot be rigid but adaptable, to reflect these differences. Already, many departments have at least unwritten guidelines that could be discussed further and captured in writing. Also apparent from the responses to the survey, software expertise and a software safety mindset exist amongst the medical physicists.

Part of the survey addressed the issue of training. Nearly 80% of the respondents chose one‐on‐one instruction, or mentoring, as a method of training. This is a common method with complex scientific type software. The need for other methods of training should be assessed by each center, based on the background of the user and the impact of the use of the software.

Associated with training, the survey examined the availability of useful documentation. Over 70% of respondents had some sort of documentation available for their software. The worrying proportion is the 25% who had little or none. However, the set of documents normally prescribed in software engineering textbooks has been called into question. Lethbridge et al.^(11)^ state that most prescribed documentation is “based on opinion and conjecture” and that “we need to better understand the various roles of software documentation”. Research, such as that by Lutters and Seaman,^(18)^ suggests useful documentation can take a number of different forms and is based on the context of the information needs. This would suggest that each medical physics department determine what documentation best fits their people, the tasks they perform, and the characteristics of the software they use.

The area of second most concern chosen by the survey respondents was “inadvertent modification” of software. In the multiple choice survey question (Part 1, Question 4) dealing with inadvertent modification, all choices were heavily used (all over 40% and one over 70%). Several other practices were listed under “other”. Guidelines can readily address this type of concern, raising awareness that various options and techniques are available to guard against lost or changed software and data.

The area of most concern chosen by the survey respondents was testing. The survey responses provided a list of testing techniques that were varied, thoughtful, and thorough. An instructional‐style of guideline might be useful, based on the existing testing expertise in the medical physics community. Scientists in general design their testing activities^(19)^ to specifically address the situation at hand. Experience in test design and a scientific mindset of “leaving no stone unturned” underlie good testing strategies for the medical physicists.

Besides listing a variety of testing techniques, the survey respondents often mentioned having someone else involved in the testing exercise. Team work is important in all scientific endeavors, and good management supports long‐term teams, good communication, and free exchange of ideas and concerns.

The conclusion from the survey is that software quality guidelines for in‐house software are seen to be useful additions to the medical physics discipline across Canada. But these guidelines must recognize the special role this software plays within this discipline. Typical of scientific software, these guidelines are better to come from within the discipline than be imposed from outside.^(16)^ The expertise is within the community to do it. Most of the list of successful software practices gleaned from other risk‐averse scientific disciplines^(10)^ are clearly apparent amongst the medical physicists who responded to the survey. The success of producing useful and useable software guidelines comes from the medical physics community educating themselves and gaining confidence in their software abilities, and management recognizing the importance of existing and credible software abilities in their staff.
